# Pre-frailty and frailty as predictors of mortality among colorectal cancer survivors: Evidence from the National Health Interview Survey (1997–2018)

**DOI:** 10.1097/MD.0000000000047702

**Published:** 2026-02-13

**Authors:** Hongyin Zhou, Wen Li, Siqi Liu, Hui Zhang, Yaxin Huang, Yonggang Hu

**Affiliations:** a Department of Clinical Laboratory, Zigong First People’s Hospital, Zigong, Sichuan, China; b Department of Clinical Laboratory, Panzhihua Central Hospital, Panzhihua, Sichuan, China; c Department of Clinical Laboratory, Gaoxin Tumor Hospital, Zigong, Sichuan, China; d Department of Transfusion, Beijing Anzhen Nanchong Hospital of Capital Medical University & Nanchong Central Hospital, Nanchong, Sichuan, China; e Department of Clinical Laboratory, People’s Hospital of Naxi District, Luzhou, Sichuan, China.

**Keywords:** colorectal cancer survivors, FRAIL scale, frailty, mortality, survival analysis

## Abstract

Frailty is a multidimensional syndrome associated with increased vulnerability to adverse health outcomes, particularly among older adults. Its relevance in cancer survivorship is increasingly recognized, yet the prognostic implications of frailty and pre-frailty among colorectal cancer survivors remain poorly defined. We conducted a prospective survival analysis using data from the 1997 to 2018 National Health Interview Survey, linked to mortality outcomes through December 31, 2019, via the National Death Index. Frailty status was determined using a modified fatigue, resistance, ambulation, illnesses, and low body mass index scale and categorized as robust (score = 0), pre-frail (score = 1–2), or frail (score = 3–5). Cox proportional hazards models were used to estimate hazard ratios (HRs) for all-cause mortality by frailty status, adjusting for demographic, socioeconomic, and clinical variables. Subgroup analyses were conducted by age and sex. Among 4052 colorectal cancer survivors, 70.2% were robust, 12.4% pre-frail, and 17.4% frail. Frailty and pre-frailty were more prevalent among survivors than among cancer-free participants (6.5% frail; 5.0% pre-frail). In fully adjusted models, pre-frail and frail survivors had significantly higher risks of all-cause mortality compared to robust individuals (HR for pre-frail, 1.44; 95% confidence interval, 1.21–1.71; *P* < .001; HR for frail, 2.19; 95% confidence interval, 1.89–2.56; *P* < .001). These associations persisted across age and sex subgroups, although they were attenuated in younger adults and in men for pre-frailty. Kaplan–Meier curves demonstrated significantly reduced survival across increasing frailty categories. Frailty and pre-frailty are common among colorectal cancer survivors and are independently associated with increased risk of all-cause mortality. Frailty assessment may help identify vulnerable colorectal cancer survivors and inform risk stratification in survivorship care planning.

## 1. Introduction

Frailty, a multidimensional syndrome characterized by decreased physiologic reserve and diminished capacity to cope with stressors, has emerged as a key determinant of adverse health outcomes in aging populations.^[1-4]^ Defined by impairments across multiple domains – including energy, mobility, strength, and comorbidity burden – frailty is associated with functional decline, hospitalization, institutionalization, and premature death.^[5-8]^ In recent years, there has been growing recognition of the importance of frailty in the context of oncology, particularly in survivorship care. Cancer survivors, especially those diagnosed at older ages (typically defined as 60 years or older in epidemiologic studies), are at elevated risk of developing or exacerbating frailty due to the combined effects of aging, cancer biology, and treatment-related toxicities.^[9-12]^

Colorectal cancer is the third most commonly diagnosed malignancy in the United States and a leading cause of cancer-related mortality. Advances in early detection and treatment have substantially improved survival, resulting in a rapidly expanding population of long-term colorectal cancer survivors.^[12-14]^ However, survivorship is often accompanied by late treatment effects, multimorbidity, and functional limitations – factors that may predispose individuals to frailty, which in turn may negatively impact long-term outcomes such as disability progression, hospitalizations, and all-cause mortality.^[15-17]^ Despite its clinical relevance, the prognostic role of frailty in colorectal cancer survivors remains inadequately characterized.

Previous studies assessing frailty in oncology populations have largely relied on clinical trial cohorts, hospital-based samples, or frailty measures not validated for population-level use.^[18-20]^ Furthermore, few investigations have examined the full frailty spectrum – including robust, pre-frail, and frail states – or compared frailty patterns between cancer survivors and individuals without cancer. The distinction between pre-frailty and frailty is particularly important, as pre-frailty represents a potentially reversible stage in the frailty trajectory.^[21-25]^ Clarifying the prognostic implications of these intermediate states could offer critical opportunities for early intervention and risk stratification in survivorship care.

To address these knowledge gaps, we utilized data from the National Health Interview Survey (NHIS), a nationally representative survey of US adults, linked to long-term mortality outcomes from the National Death Index. We applied a modified version of the fatigue, resistance, ambulation, illnesses, and low body mass index (FRAIL) scale, a simple and validated screening tool suitable for large-scale population studies, to classify frailty status among colorectal cancer survivors. Our objectives were threefold: to characterize the distribution of frailty among colorectal cancer survivors compared with cancer-free individuals; to evaluate the association between frailty status and all-cause mortality; and to examine whether these associations vary by age and sex. This study provides novel, population-based evidence on the prognostic significance of frailty in colorectal cancer survivorship and has potential implications for personalized survivorship care and long-term health planning.

## 2. Materials and methods

### 2.1. Study design and population selection

We conducted a longitudinal survival analysis using baseline data from the NHIS, a nationally representative cross-sectional survey, with prospective follow-up for all-cause mortality through linkage to the National Death Index. The NHIS is an ongoing, nationally representative, cross-sectional survey administered by the National Center for Health Statistics (NCHS) that gathers information on health status, health-related behaviors, and sociodemographic characteristics of the noninstitutionalized US civilian population.

From 1997 to 2018, a total of 6,71,696 adult participants were identified from the NHIS. We excluded individuals with missing data on frailty status (N = 28,461), covariates (N = 47,238), or mortality outcomes (N = 5742), as well as those who reported a history of cancer other than colorectal cancer (N = 673). After these exclusions, 5,89,582 participants were included in the final analytic sample.

**Figure 1.**
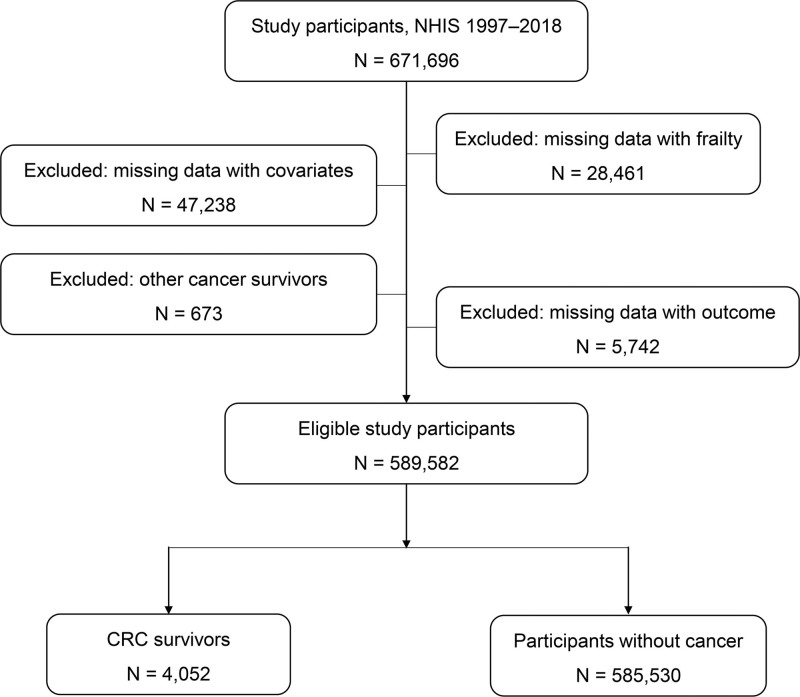
Flow chart of study participants selection. CRC = colorectal cancer, NHIS = National Health Interview Survey.

Among these, 4052 were colorectal cancer survivors, and 5,85,530 participants had no history of cancer. A detailed flow diagram of participant selection is shown in Figure 1.

### 2.2. Frailty assessment using the FRAIL scale

Frailty status was determined using a modified version of the FRAIL scale, a well-established screening tool originally proposed by the Geriatric Advisory Panel of the International Society for Nutrition and Aging.^[26,27]^ In this analysis, we operationalized frailty using self-reported data collected from the NHIS between 1997 and 2018. The FRAIL scale includes 5 domains: fatigue, resistance, ambulation, comorbid illness burden, and low body mass index (BMI).^[28]^

Fatigue was identified based on participants’ responses to NHIS survey items assessing how often they felt unusually tired or lacking in energy over a specified timeframe. Frequent or sustained fatigue was scored as 1, whereas minimal or no fatigue was scored as 0.

Resistance was evaluated through questions on the ability to climb 12 stairs without help or the use of mobility aids. Ambulation was assessed by inquiring whether participants had difficulty walking 100 yards on a flat surface (equivalent to the length of a football field or city block) without assistance. For both domains, the presence of any reported difficulty was scored as 1; absence of difficulty was scored as 0.

The illness component reflected multimorbidity. Participants were assigned a score of 1 if they reported 5 or more chronic health conditions from a list of 12 physician-diagnosed diseases: angina, arthritis, asthma, anxiety disorder, chronic obstructive pulmonary disease, coronary heart disease, dementia, diabetes, myocardial infarction, hyperlipidemia, hypertension, and stroke. Reporting fewer than 5 conditions resulted in a score of 0.

Low BMI was defined as a BMI below 18.5 kg/m^2^. Participants meeting this criterion received a score of 1; all others were scored as 0.

Scores across the 5 components were summed to yield a total frailty score ranging from 0 to 5. Based on established cutoffs, participants were categorized as frail (score 3–5), pre-frail (score 1–2), or robust (score 0).^[27]^

Frailty status reflects participants’ self-reported health and functional status at the time of the NHIS interview, which occurred after their initial cancer diagnosis and may represent varying intervals since treatment.

### 2.3. Ethical considerations

The NHIS is conducted by the NCHS and has been approved by the NCHS Research Ethics Review Board. All participants provided informed consent at the time of the interview. The current study used publicly available, de-identified NHIS data linked with the National Death Index. As such, this secondary analysis was deemed exempt from institutional review board oversight.

### 2.4. Covariates

We assessed a range of covariates using data from the NHIS, including age, sex, race and ethnicity, educational attainment, health insurance status, marital status, geographic region, history of depression, time since cancer diagnosis, and number of cancer diagnoses. The NHIS is an annual, nationally representative, cross-sectional survey conducted by the NCHS to monitor the health status of the non-institutionalized civilian population in the United States.

Participants reported their age in years at the time of the household interview and identified their sex as either male or female. Race and Hispanic ethnicity were determined through self-report, with participants selecting 1 or more racial groups and indicating whether they were of Hispanic or Latino origin. For analytical purposes, race/ethnicity was grouped into 4 categories: White, Black, Asian, and Other.

Educational level was based on the highest degree or level of schooling completed and was classified as less than high school, high school graduate, or more than high school. Health insurance coverage was assessed at the time of interview and included private insurance, Medicare, Medicaid, or other government-sponsored plans; responses were dichotomized as insured or uninsured. Marital status was categorized as married or unmarried based on self-reported current relationship status.

Participants’ geographic location was classified into one of 4 regions – Northeast, Midwest, South, and West – according to the US Census Bureau definitions used by the NHIS. Depression was determined by self-report of a physician or health professional diagnosis of depression, consistent with the approach used in prior NHIS-based epidemiologic research.

#### 2.4.1. Assessment of cancer history

Information on cancer history was based on self-reported responses in the NHIS. Participants who responded “yes” to the question “Have you ever been told by a doctor or other health professional that you had cancer or a malignancy of any kind?” were classified as having a history of cancer. *Time since cancer diagnosis* was calculated as the difference between the year of interview and the self-reported year of the first cancer diagnosis, and was categorized as <2 years or≥2 years. The *number of cancer diagnoses* was derived from the total number of different cancer types reported by each participant, and was classified as 1 or ≥2. Both variables were included as categorical covariates in multivariable analyses to account for the potential confounding effect of cancer history.

These covariates were selected due to their established relevance in health outcomes research, and the use of standardized NHIS measures enhances the comparability and validity of our findings.

### 2.5. All-cause mortality

Mortality follow-up for NHIS participants was achieved through linkage to the National Death Index, covering deaths through December 31, 2019. This linkage, conducted by the NCHS as part of its Data Linkage Program, employed probabilistic record-matching techniques to connect NHIS survey data with National Death Index death certificate records. The process enabled accurate determination of vital status and cause-specific mortality.

Strict confidentiality procedures were observed throughout the linkage process. In the publicly available linked mortality files (LMF), data perturbation methods were applied to minimize reidentification risk. For a subset of participants, synthetic values were substituted for certain variables, such as follow-up time or cause of death. Importantly, vital status information was preserved without alteration.

Using the NHIS LMF, we conducted analyses to evaluate the association between frailty status and all-cause mortality. This approach integrated prospectively collected health and demographic data with long-term mortality follow-up, allowing for a robust examination of mortality risk in relation to frailty.

### 2.6. Statistical analysis

All statistical analyses were conducted using SAS software, version 9.4 (SAS Institute Inc., Cary) and R software, version 4.3.1 (R Foundation for Statistical Computing, Vienna, Austria; https://www.R-project.org). Descriptive statistics were used to summarize the baseline characteristics of participants. Means and standard deviations (SDs) were reported for continuous variables, while categorical variables were presented as frequencies and percentages.

To assess differences in baseline characteristics across frailty categories (robust, pre-frail, and frail), we used 1-way analysis of variance for continuous variables and Pearson’s chi-square (χ^2^) test for categorical variables. Post hoc pairwise comparisons were performed as needed to identify specific group differences.

Survival probabilities were estimated using the Kaplan–Meier (KM) method, and differences in survival across frailty categories were evaluated using the log-rank test. Median survival times and 95% confidence intervals (CIs) were calculated where appropriate.

To examine the association between frailty status and all-cause mortality, we employed Cox proportional hazards regression models. Hazard ratios (HRs) and 95% CIs were estimated for pre-frail and frail participants, using the robust group as the reference. Two models were constructed: an age- and sex-adjusted model, and a fully adjusted multivariable model incorporating additional covariates, including race/ethnicity, educational level, health insurance status, marital status, region, self-reported depression, time since cancer diagnosis, and number of cancer diagnoses. The proportional hazards assumption was tested using Schoenfeld residuals and was not violated.

All statistical tests were 2-sided, and a *P*-value of <.05 was considered to indicate statistical significance. No imputation was performed for missing data, as the analytic sample was restricted to individuals with complete information on all variables of interest.

## 3. Results

### 3.1. Study population characteristics

A total of 4052 adults with self-reported colorectal cancer were included in the analytic sample, comprising 2847 (70.3%) robust individuals, 501 (12.4%) pre-frail individuals, and 704 (17.3%) frail individuals (Table 1). The mean age increased across frailty categories, from 69.1 years (SD = 12.6) in the robust group to 73.0 years (SD = 10.4) in the pre-frail group and 73.4 years (SD = 11.6) in the frail group (*P* < .001).

**Table 1:** Baseline characteristics of colorectal cancer patients according to frailty status, NHIS 1997 to 2018.

Characteristics	Robust 2847 (70.3%)	Pre-frail 501 (12.4%)	Frail 704 (17.3%)	*P*-value*
Age, yr	69.1 (12.6)	73.0 (10.4)	73.4 (11.6)	<.001
Age group				<.001
<60 yr old	700 (24.6%)	80 (16.0%)	60 (8.5%)	
≥60 yr old	2147 (75.4%)	421 (84.0%)	644 (91.5%)	
Sex, %				<.001
Women	1419 (49.8)	230 (45.9)	404 (57.4)	
Men	1428 (50.2)	271 (54.1)	300 (42.6)	
Race/ethnicity, %				<.001
White	1595 (56.0)	295 (58.9)	134 (19.0)	
Black	243 (8.5)	43 (8.6)	32 (4.5)	
Asian	10 (0.4)	1 (0.2)	0 (0.0)	
Other	999 (35.1)	162 (32.3)	538 (76.5)	
Education level, %				<.001
<High school	1304 (45.8)	256 (51.1)	397 (56.4)	
High school graduate	575 (20.2)	98 (19.6)	156 (22.2)	
>High school	968 (34.0)	147 (29.3)	151 (21.4)	
Health insurance, %				.004
Yes	2813 (98.8)	490 (97.8)	684 (97.2)	
No	34 (1.2)	11 (2.2)	20 (2.8)	
Marital status, %				<.001
Married	1209 (42.5)	194 (38.7)	218 (31.0)	
Unmarried	1638 (57.5)	307 (61.3)	486 (69.0)	
Region, %				.013
Northeast	499 (18.3)	99 (20.2)	110 (17.1)	
Midwest	680 (24.9)	118 (24.2)	187 (29.0)	
South	979 (35.8)	173 (35.5)	251 (38.9)	
West	573 (21.0)	98 (20.1)	97 (15.0)	
Depression, %				<.001
No	2772 (97.4)	471 (94.0)	653 (92.8)	
Yes	75 (2.6)	30 (6.0)	51 (7.2)	
Time since cancer diagnosis				<.001
<2 yr	390 (13.7)	94 (18.7)	177 (25.1)	
≥2 yr	2457 (86.3)	407 (81.3)	527 (74.9)	
Number of cancer diagnoses				<.001
1	2249 (79.0)	418 (83.5)	621 (88.2)	
≥2	598 (21.0)	83 (16.5)	83 (11.8)	

Values are means (SDs) for continuous variables and percentages for categorical variables.

* Group differences were assessed using analysis of variance (ANOVA) for continuous variables and the chi-square test for categorical variables.

ANOVA = analysis of variance, NHIS = National Health Interview Survey, SD = standard deviation.

Differences in sociodemographic characteristics were evident across frailty groups. Frail individuals were more likely to be female (57.4%) than those who were pre-frail (45.9%) or robust (49.8%; *P* < .001). The distribution of race and ethnicity varied significantly, with a markedly higher proportion of individuals identifying as “Other” among the frail group (76.5%) compared to the robust (35.1%) and pre-frail (32.3%) groups (*P* < .001).

Educational attainment was inversely associated with frailty: 56.4% of frail individuals had less than a high school education, compared with 51.1% and 45.8% in the pre-frail and robust groups, respectively (*P* < .001). The proportion of participants without health insurance was higher among frail individuals (2.8%) than among those who were pre-frail (2.2%) or robust (1.2%; *P* = .004).

Marital status differed significantly by frailty status. The proportion of unmarried individuals was highest among those categorized as frail (69.0%), followed by pre-frail (61.3%) and robust (57.5%) participants (*P* < .001). Geographic distribution also varied, with a greater proportion of frail individuals residing in the South and Midwest (*P* = .013).

Finally, the prevalence of self-reported depression increased across the frailty spectrum, from 2.6% in the robust group to 6.0% in the pre-frail group and 7.2% in the frail group (*P* < .001).

### 3.2. Comparison of frailty status between colorectal cancer survivors and cancer-free participants

**Figure 2.**
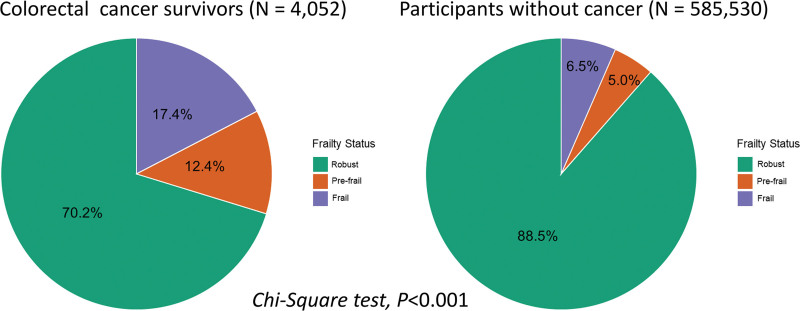
Distribution of frailty status among colorectal cancer survivors and cancer-free participants. This figure illustrates the distribution of frailty status among colorectal cancer survivors (N = 4052) and cancer-free participants (N = 5,85,530) in the National Health Interview Survey (NHIS), 1997 to 2018. Frailty was assessed using a modified FRAIL scale and categorized as robust (score = 0), pre-frail (score = 1–2), and frail (score = 3–5). Among colorectal cancer survivors, 70.2% were classified as robust, 12.4% as pre-frail, and 17.4% as frail. In contrast, among participants without a history of cancer, 88.5% were robust, 5.0% were pre-frail, and 6.5% were frail. The difference in frailty distribution between the 2 groups was statistically significant (*P* < .001 by chi-square test). FRAIL = fatigue, resistance, ambulation, illnesses, and low body mass index scale, NHIS = National Health Interview Survey.

Frailty status differed significantly between colorectal cancer survivors and individuals without a history of cancer (*P* < .001 by chi-square test), as illustrated in Figure 2. Among survivors of colorectal cancer, 70.2% were classified as robust, 12.4% as pre-frail, and 17.4% as frail. In contrast, the distribution of frailty among cancer-free participants showed a higher proportion of robust individuals (88.5%) and markedly lower proportions of both pre-frail (5.0%) and frail (6.5%) individuals. These differences were illustrated in Figure 2.

### 3.3. Survival probability across different frailty statuses in colorectal cancer survivors and cancer-free participants

**Figure 3.**
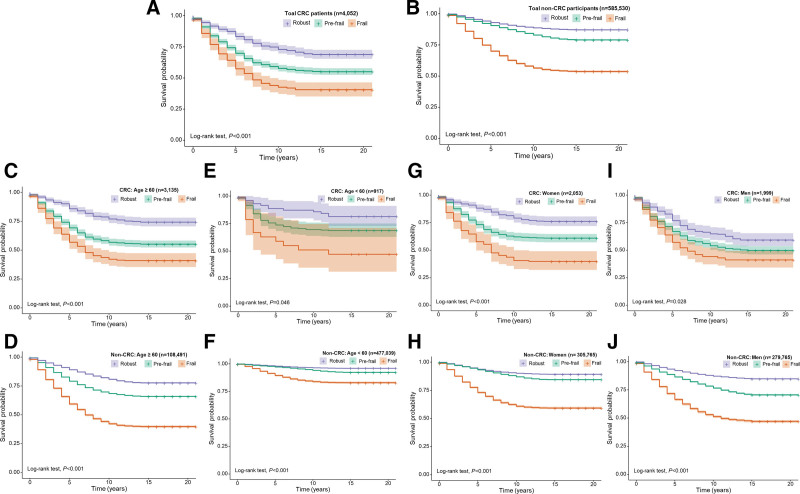
Kaplan–Meier survival curves for all-cause mortality by frailty status among colorectal cancer survivors and cancer-free participants. Survival probabilities were compared across frailty categories (robust, pre-frail, and frail) in both colorectal cancer survivors and cancer-free individuals using data from NHIS 1997 to 2018. Panel (A) shows overall colorectal cancer survivors, while panel (B) shows overall cancer-free participants. Panels (C, D) present results for individuals aged 60 years or older in the survivor and cancer-free groups, respectively. Panels (E, F) show results for participants younger than 60 years. Panels (G, H) display the survival curves for female participants in the survivor and cancer-free groups, respectively, and panels (I, J) show the corresponding results for male participants. In all subgroups, survival probability declined with increasing frailty status. The separation between frailty categories was more pronounced among colorectal cancer survivors than among cancer-free individuals. Log-rank tests were used to assess statistical significance.

KM survival curves demonstrated significant differences in all-cause mortality across frailty categories among colorectal cancer survivors (*P* < .001 by log-rank test; Figure 3A). Robust individuals exhibited the highest survival probability over time, followed by those categorized as pre-frail and frail.

The restricted mean survival time up to 20 years of follow-up among colorectal cancer survivors was 13.72 years, based on the KM survival function.

This pattern remained consistent in subgroup analyses. Among survivors aged 60 years or older, survival curves continued to diverge significantly by frailty status (*P* < .001) and closely mirrored those observed in the overall cohort (Fig. 3C). Similarly, among female survivors, survival probabilities differed markedly across frailty groups (*P* < .001), showing a trend comparable to that of the overall study population and the older subgroup (Fig. 3G).

Among survivors younger than 60 years, the survival curves also differed significantly by frailty status (*P* = .046); however, wide confidence intervals reflected smaller sample sizes and greater uncertainty in the estimates (Fig. 3E). In male survivors, the association between frailty status and survival remained significant (*P* = .028), though the separation between frailty groups was less pronounced than in other subgroups (Fig. 3I).

For comparison, we also examined survival probabilities among cancer-free participants. As shown in Figure 3B, D–J, frailty was associated with significantly reduced survival in both populations, but the magnitude of survival differences across frailty categories was greater among colorectal cancer survivors than among cancer-free participants.

### 3.4. Prospective analysis of frailty and all-cause mortality

Frailty status was significantly associated with all-cause mortality among colorectal cancer survivors (Table 2). In the fully adjusted model, individuals classified as pre-frail had a 44% higher risk of death compared with those who were robust (hazard ratio [HR], 1.44; 95% confidence interval [CI], 1.21–1.71; *P* < .001), while frail individuals had more than double the mortality risk (HR, 2.19; 95% CI, 1.89–2.56; *P* < .001). These associations remained robust after adjustment for age, sex, race/ethnicity, education level, insurance status, marital status, region, depression, time since cancer diagnosis, and number of cancer diagnoses.

**Table 2:** Association between frailty status and all-cause mortality among colorectal cancer patients, NHIS 1997 to 2018.

Frailty status	Age- and sex-adjusted model*			Multivariate adjusted model†		
	HR	95% CI	*P*-value	HR	95% CI	*P*-value
Total colorectal cancer patients						
Robust	Reference	Reference		Reference	Reference	
Pre-frail	1.51	1.27–1.80	<.001	1.44	1.21–1.71	<.001
Frail	2.26	1.98–2.77	<.001	2.19	1.89–2.56	<.001
≥ 60 yr old						
Robust	Reference	Reference		Reference	Reference	
Pre-frail	1.50	1.25–1.79	<.001	1.45	1.21–1.74	<.001
Frail	2.54	2.31–2.80	<.001	2.47	2.14–2.78	<.001
<60 yr old						
Robust	Reference	Reference		Reference	Reference	
Pre-frail	2.01	1.13–3.57	.017	1.81	1.01–3.25	.046
Frail	2.47	0.88–4.02	.285	2.25	0.80–3.98	.261
Women						
Robust	Reference	Reference		Reference	Reference	
Pre-frail	1.70	1.32–2.19	<.001	1.62	1.25–2.10	<.001
Frail	2.62	2.13–3.05	<.001	2.55	2.01–2.94	<.001
Men						
Robust	Reference	Reference		Reference	Reference	
Pre-frail	1.26	1.00–1.59	.053	1.21	0.95–1.53	.118
Frail	2.13	1.87–2.48	<.001	2.07	1.80–2.39	<.001

CI = confidence interval, HR = hazards ratio, NHIS = National Health Interview Survey.

* Cox regression model adjusted for age and sex.

† Cox regression model adjusted for age, sex, race/ethnicity, education level, health insurance, marital status, region, depression, time since cancer diagnosis, and number of cancer diagnoses.

Subgroup analyses revealed consistent patterns across age and sex strata. Among participants aged 60 years or older, both pre-frailty (HR, 1.45; 95% CI, 1.21–1.74; *P* < .001) and frailty (HR, 2.47; 95% CI, 2.14–2.78; *P* < .001) were associated with significantly increased mortality. In contrast, among those younger than 60 years, pre-frailty remained significantly associated with higher mortality (HR, 1.81; 95% CI, 1.01–3.25; *P* = .046), whereas frailty did not reach statistical significance (HR, 2.25; 95% CI, 0.80–3.98; *P* = .261), likely due to limited sample size and wide confidence intervals.

Sex-stratified analyses demonstrated a stronger association between frailty and mortality among women. In fully adjusted models, pre-frailty (HR, 1.62; 95% CI, 1.25–2.10; *P* < .001) and frailty (HR, 2.55; 95% CI, 2.01–2.94; *P* < .001) were both significantly associated with increased mortality in women. Among men, frailty was also significantly associated with mortality (HR, 2.07; 95% CI, 1.80–2.39; *P* < .001), but pre-frailty was not (HR, 1.21; 95% CI, 0.95–1.53; *P* = .118), suggesting potential sex-based differences in frailty’s prognostic value.

## 4. Discussion

In this nationally representative study of over 4000 colorectal cancer survivors, we found that both frailty and pre-frailty were significantly more prevalent in survivors than in cancer-free individuals, and both conditions were independently associated with increased all-cause mortality. Among survivors, nearly 1 in 5 met criteria for frailty, and an additional 12.4% were classified as pre-frail – more than twice the prevalence observed in the cancer-free population. These findings underscore the high burden of physiological vulnerability in colorectal cancer survivors and suggest that frailty may play a central role in shaping long-term survival outcomes, specifically the risk of all-cause mortality, in this growing population.

Notably, when comparing colorectal cancer survivors with cancer-free participants, frailty was associated with reduced survival in both groups, but the survival gap across frailty categories was more pronounced among survivors. This finding suggests that colorectal cancer history may exacerbate the adverse prognostic impact of frailty, potentially through persistent treatment-related toxicities, comorbidities, and accelerated biological aging. These results highlight the importance of routine frailty assessment not only in the general population but especially in cancer survivorship care.

Our study adds to a small but growing body of literature that highlights the importance of frailty in cancer survivorship. While prior studies have demonstrated that frailty predicts poor short-term outcomes – such as treatment-related complications, hospitalizations, or early functional decline – in older cancer patients undergoing treatment, few have examined its long-term prognostic implications among survivors in the general population.^[12,25]^ By using data from the NHIS linked to mortality outcomes, we were able to evaluate these associations in a large, community-based sample, thereby enhancing the generalizability and public health relevance of our findings. Furthermore, we leveraged a validated, multidimensional frailty tool (the FRAIL scale) to capture functional, clinical, and physiological domains, allowing for a comprehensive characterization of frailty status.

The observed stepwise association between frailty severity and mortality suggests that even early frailty manifestations carry prognostic significance. Pre-frail individuals had a 44% increased risk of death compared with their robust counterparts, while frail individuals had more than a twofold risk. These associations remained robust after adjustment for key demographic, socioeconomic, and health-related confounders.^[29-31]^ Importantly, our findings were consistent across subgroups, including women and adults aged 60 years or older, although estimates were less precise among younger survivors due to smaller sample sizes.

In addition to clinical sequelae, psychosocial and demographic factors may also contribute to frailty vulnerability in colorectal cancer survivors. Our finding that frailty was disproportionately more common among women aligns with previous research showing that women often experience longer survival but with higher levels of disability and chronic illness, which may increase frailty risk. Women may also be more likely to report symptoms such as fatigue or reduced mobility – key components of frailty assessment. Similarly, depression has been linked to frailty through pathways including systemic inflammation, hormonal dysregulation, physical inactivity, and malnutrition. Individuals with limited social support may face increased psychosocial stress, loneliness, and reduced access to healthcare services or health-promoting behaviors, all of which may exacerbate physiologic decline. These findings underscore the multidimensional nature of frailty and highlight the need to consider both medical and social determinants of health in survivorship care.

Several additional factors may contribute to the excess burden of frailty observed among colorectal cancer survivors. Cancer-related fatigue, persistent gastrointestinal symptoms, and chemotherapy-induced neuropathy may collectively impair physical functioning long after treatment completion.^[32,33]^ These chronic effects can limit mobility, reduce exercise capacity, and promote sedentary behavior, all of which accelerate frailty progression.^[34]^ Survivors often face complex medication regimens and multimorbidity, which may further compromise physiologic reserve.^[35]^ In addition, disparities in access to rehabilitation, nutritional support, and psychosocial services may exacerbate frailty risk, particularly among socioeconomically disadvantaged individuals.^[36]^ Our findings that frailty was more common in participants with lower educational attainment, lack of insurance, and depression highlight the convergence of clinical and social vulnerability in shaping long-term survivorship trajectories.

These findings have important implications for survivorship care. As life expectancy improves among colorectal cancer patients, survivorship strategies must move beyond surveillance for recurrence and second primaries to encompass functional health and aging-related vulnerability. Frailty screening using simple instruments such as the FRAIL scale may help identify survivors at high risk for functional decline, hospitalization, or premature death. Importantly, pre-frailty – often overlooked in clinical practice – represents a potentially reversible state. Interventions such as tailored exercise programs, nutritional support, and multimorbidity management have shown promise in reversing or attenuating frailty progression.^[37-41]^ Embedding frailty screening into survivorship care plans – especially for older adults, women, and those with multiple chronic conditions – could help clinicians personalize surveillance intensity, prioritize supportive services, and coordinate multidisciplinary care. Research into scalable models of frailty mitigation, including community-based programs and geriatric co-management, is warranted to support the translation of frailty-informed care into oncology practice.

Several mechanisms may explain the observed associations between frailty status and all-cause mortality. Cancer diagnosis and treatment can accelerate biological aging through pathways such as inflammation, hormonal dysregulation, mitochondrial dysfunction, and muscle catabolism.^[4,42-45]^ Survivors may also experience long-term sequelae of chemotherapy, radiation, or surgical interventions that compromise mobility, nutritional status, and psychological well-being.

Our study has several strengths, including the use of a large, nationally representative dataset, a validated frailty instrument, and long-term mortality follow-up. The analytic approach accounted for a wide range of potential confounders, enhancing internal validity. Nevertheless, several limitations should be acknowledged. Frailty was assessed at a single time point using self-reported data, which may be subject to measurement error or misclassification. Although the FRAIL scale is well-suited for population-based research, it may not capture more nuanced or subclinical manifestations of frailty. Furthermore, cause-specific mortality could not be evaluated due to data constraints in the publicly available NHIS LMF. Finally, information on cancer stage, tumor characteristics, and treatment history was not available in the NHIS dataset, which may lead to residual confounding. Although we adjusted for time since diagnosis and number of cancer diagnoses, we could not account for differences in cancer severity at baseline.

## 5. Conclusions

In conclusion, frailty and pre-frailty are common among colorectal cancer survivors and are independently associated with increased risk of all-cause mortality. These findings support the potential value of frailty assessment in survivorship care planning. Further research is warranted to explore whether targeted interventions can mitigate these risks and improve survivorship outcomes.

## Acknowledgments

We thank the Director and staff of the Department of Clinical Laboratory, People’s Hospital of Naxi District, for their valuable assistance and full support during the conduct of this study.

## Author contributions

**Conceptualization:** Hongyin Zhou, Wen Li, Yonggang Hu.

**Data curation:** Hongyin Zhou.

**Methodology:** Yaxin Huang.

**Software:** Hui Zhang, Yaxin Huang.

**Visualization:** Hui Zhang, Yaxin Huang.

**Writing – original draft:** Hongyin Zhou, Siqi Liu.

**Writing – review & editing:** Yonggang Hu.
